# Socio-Ecological Influences on Adolescent (Aged 10–17) Alcohol Use and Unhealthy Eating Behaviours: A Systematic Review and Synthesis of Qualitative Studies

**DOI:** 10.3390/nu11081914

**Published:** 2019-08-15

**Authors:** Stephanie Scott, Wafa Elamin, Emma L. Giles, Frances Hillier-Brown, Kate Byrnes, Natalie Connor, Dorothy Newbury-Birch, Louisa Ells

**Affiliations:** 1 School of Humanities and Social Sciences, Teesside University, Middlesbrough TS1 3BA, UK; 2 Institute of Health and Society, Newcastle University, Newcastle upon Tyne NE1 4AX, UK; 3 School of Health and Social Care, Teesside University, Middlesbrough TS1 3BA, UK; 4 Department of Sport and Exercise Sciences, Durham University, Durham DH1 3HN, UK

**Keywords:** adolescent, eating, alcohol use, qualitative research, systematic review

## Abstract

Excess body weight and risky alcohol consumption are two of the greatest contributors to global disease. Alcohol use contributes directly and indirectly to weight gain. Health behaviours cluster in adolescence and track to adulthood. This review identified and synthesised qualitative research to provide insight into common underlying factors influencing alcohol use and unhealthy eating behaviours amongst young people aged 10–17. Sixty two studies met inclusion criteria. Twenty eight studies focused on alcohol; 34 focused on eating behaviours. Informed by principles of thematic analysis and meta-ethnography, analysis yielded five themes: (1) use of alcohol and unhealthy food to overcome personal problems; (2) unhealthy eating and alcohol use as fun experiences; (3) food, but not alcohol, choices are based on taste; (4) control and restraint; and (5) demonstrating identity through alcohol and food choices. Young people faced pressure, reinforced by industry, to eat and drink in very specific ways, with clear social consequences if their attitudes or behaviour were deemed unacceptable. No qualitative studies were identified with an explicit and concurrent focus on adolescent eating behaviours and alcohol consumption. Further exploratory work is needed to examine the links between food and alcohol in young people’s emotional, social and cultural lives.

## 1. Introduction

Excess body weight and heavy alcohol consumption are two of the greatest contributors to global disease burden in high-income countries [1,2]. Despite reductions in the overall prevalence of youth drinking in recent years, risky or heavy alcohol use remains the leading cause of death and disability-adjusted life years in both 15–19-year-olds and 20–24-year-olds globally [3]. Meanwhile, the number of children and adolescents (aged 2–19 years) overweight or obese in developed countries is estimated to be 24% of boys and 23% of girls [4]. Weight status and consumption of alcohol are both associated with a number of negative social, emotional and cultural outcomes during adolescence and early adulthood such as risky sexual behaviour, poor quality of life, negative effects on mental and physical health, poor educational outcomes, youth offending, and the development of alcohol-use disorders [5,6,7,8,9,10,11,12]. Further, weight gain during adolescence may to lead to disordered patterns of eating and overeating, reported to be triggered by social and emotional factors, and in particular, bullying [13,14]. Body mass index (BMI) and alcohol consumption interact, with a steeply elevated risk of liver disease observed for those with both high BMI and alcohol consumption [15]. Heavy drinking is also associated with greater waist-hip-ratio in mid-life even when taking other lifetime influences into account [16]. Adverse health behaviours such as risky drinking and unhealthy eating begin to cluster during adolescence [17], and both have been demonstrated to track into and throughout adulthood [18,19,20].

A growing body of quantitative, epidemiological data has identified that energy intake from alcohol, type of beverage, and drinking pattern (i.e., high volume, high frequency) can contribute substantially to total energy intake, and are associated with excess body weight and weight gain amongst adults [21,22,23]. Current results from the National Diet and Nutrition Survey Rolling Programme (NDNS RP, Years 1–9, 2008/9 to 2016/17) demonstrate a downward trend in the percentage of people consuming alcohol for all age groups surveyed [24]. This was statistically significant for adults aged 19–64 years (−2% per year) and for girls aged 11–18 years (−1% per year). However, for those who consumed alcohol, there was little change over time in alcohol intake as a percentage of total energy. The exception was the 11–18 years population group, where there was an average yearly decrease of 0.3 percentage points. For young people specifically (children aged 11–18), data from 2014/15 and 2015/16 of the NDNS programme highlights a mean average of 0.5% total energy intake from alcohol per day [25]. A recent systematic review and meta-analysis demonstrated that, across 22 identified studies, with 701 participants, alcoholic beverage consumption significantly increased food energy intake and total energy intake compared with a non-alcoholic comparator, suggesting that adults do not compensate appropriately for alcohol energy by eating less, and that a relatively modest alcohol dose may lead to an increase in food consumption [26]. Further, focusing specifically on young adulthood, and using pooled cross-sections of the 2008–2014 Health Survey for England and the Scottish Health Survey, Albani et al. (2018) found young adults aged 18–25 drinking the highest levels of alcohol on a single occasion were more likely to be obese than those with the lowest intake [27]; whilst Giles and Brennan (2014) highlighted that young adults make ‘trade offs’ between alcohol consumption, physical activity and eating patterns, by seeking to compensate ‘unhealthy’ behaviours with healthier ones [28]. More recently, Scott et al. (2019) identified that cultural, physical and emotional links between food and alcohol consumption were an unquestioned norm among 18–25 year-olds in the UK, with young adults calculating risks to weight, appearance and social status rather than to long-term health (in press). The latter study remains the only study to date to utilise qualitative data to examine the deeply interconnected nature of eating and drinking behaviour.

A number of influences on diet and drinking behaviours in young people (those under the age of 18) have been identified, and appear to cut across both behaviours. These include food and alcohol environments (including commercial factors such as urban space, price, promotion, and access), sometimes described as the ‘foodscape’ or ‘obesogenic’ and ‘intoxigenic’ environments, peers, family, and socio-economic status [29,30,31,32,33,34,35,36,37,38,39]. Further, there are emotional, social and symbolic benefits for young people to participating in both unhealthy eating and alcohol consumption practices for young people such as perceptions of pleasure, distinction and identity, or social status [40,41,42,43]. For example, a recent UK longitudinal study of young adults found that, contrasting with a recalled lack of concern in mid-adolescence, body-consciousness and weight-related concern generally increased around the time of school-leaving (traditionally aged 16) [44]. The authors suggest that this change resulted at least in part from increased autonomy and control over their own diet and the acknowledgement of health as personal responsibility.

Nevertheless, whilst extensive bodies of qualitative literature explore the influences on young people’s eating practices and alcohol consumption, respectively, to our knowledge, no qualitative studies explicitly focus on how young people’s eating and drinking behaviours interact and intertwine. This systematic review and qualitative synthesis aimed to address this evidence gap by bringing together two separate bodies of qualitative research evidence to examine young people’s (aged 10–17) perspectives on socio-cultural, interpersonal and structural influences upon unhealthy eating behaviours or alcohol use. Most existing reviews focusing on health behaviours answer a very specific question, such as alcohol industry efforts to influence alcohol marketing policy [45] or barriers and enablers of healthy eating in young adulthood [46]. Therefore, it was not deemed appropriate to synthesise from these reviews as a ‘review of reviews’. Instead, our intention was to capitalise on what is known from independent streams of research, enabling analysis and comparison across two associated fields of study. Thus, our primary review question is to derive socio-cultural, inter-personal and structural factors from a systematic review and synthesis of qualitative literature that might influence young people aged 10–17 among whom risky drinking and unhealthy eating co-occur, and subsequently, utilise review findings to develop a model structure of common underlying influences which cut across both behaviours in this population group.

## 2. Materials and Methods

The study protocol was pre-registered (Ref: CRD42017060624) [47] in compliance with the ‘Preferred Reporting Items for Systematic Reviews and Meta-Analyses’ (PRISMA) statement [48]. Whilst the Joanna Briggs Institute (JBI) approach was not adopted, and this review was not registered as a JBI review, it was carried out using data extraction and quality appraisal tools designed by JBI.

### 2.1. Eligibility Criteria

The following studies were eligible for inclusion: studies that reported primary data of any qualitative design and which explored the views of young people (aged 10–17 inclusive) on factors which shape their eating behaviours or alcohol consumption. Mixed method studies were considered eligible if findings from qualitative study components were reported in full and were distinguishable from other findings. We included studies published in English, from 2006 onwards. This date limit reflected limited time/resources for the review. Age criteria was determined using the age range cited in the study or mean age at interview. For longitudinal studies, this was the age at recruitment and/or first interview. If results were analysed separately for groups of different ages, and studies included younger children or participants aged 18 or more, only data relating to those aged 10–17 were extracted. If these data were not easily distinguished from other findings, the study was excluded. Unpublished data, abstracts, conference proceedings and studies that did not include primary evaluation data (e.g., protocols, reviews, editorials) were excluded from the review. Studies were excluded if they: (a) sought and reported the views of others (e.g., parents) rather than young people themselves; (b) analysed texts alone (e.g., discourse analysis); (c) used self-report or researcher-administered surveys, including those analysing data from open-ended questions. We also placed restrictions upon the study population, and studies were excluded if participants: (1) required specialist treatment for alcohol dependency or weight loss and gain; and (2) were pregnant or breastfeeding adolescent women whose current eating pattern was time-limited and not reflective of usual diet behaviours.

### 2.2. Search Strategy

In accordance with the SPIDER tool [49], the search strategy was split into five core concepts. This search strategy is documented in Supplementary Table S1. Nine electronic databases (MEDLINE, Scopus, PsychINFO, Sociological Abstracts (via ProQuest social science premium collection), CINAHL, ERIC, IBSS (via ProQuest social science premium collection), and Web of Science Core Collection) were searched from January 2006 to October 2017, using appropriate thesaurus headings and title or abstract keywords. Studies retrieved included Online First articles. Electronic database searches were supplemented by searches of Google Scholar, checking reference lists cited by included studies and checking reference lists already held by the study team.

### 2.3. Study Selection and Data Extraction

**Figure 1:**
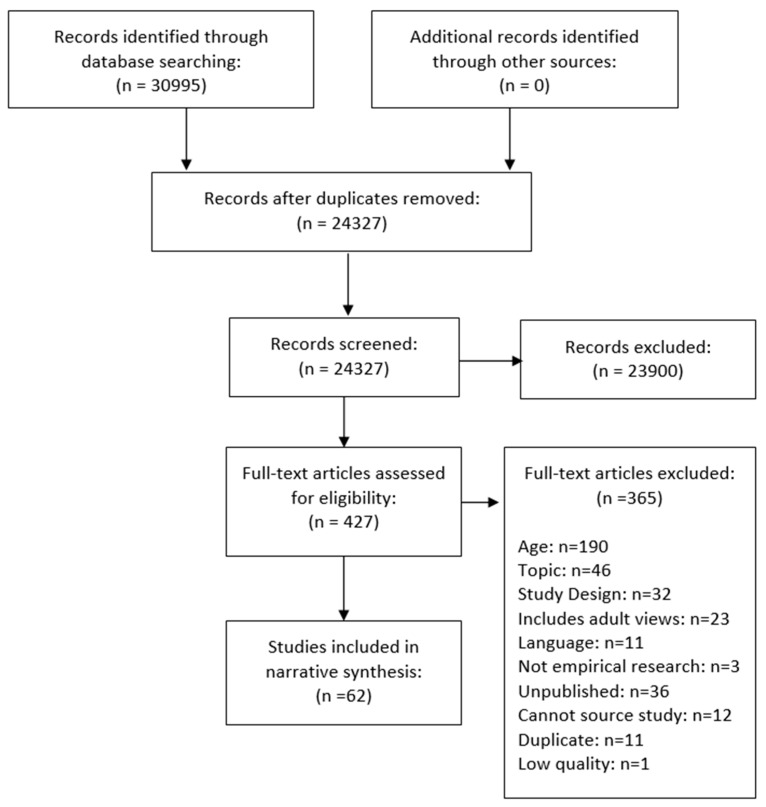
Flow chart showing study selection process.

Figure 1 provides a visual representation of the methodological process, according to the PRISMA framework [48]. The title and abstract of all records retrieved were downloaded to Endnote X7 and independently screened by five reviewers (SS, ELG, DNB, KB, NC), with full-text copies of potentially relevant papers retrieved for in-depth review against the inclusion criteria. The JBI Qualitative Assessment and Review Instrument (QARI) was adapted to an Excel spreadsheet to facilitate data extraction, with JBI SUMARI (JBI, Adelaide, SA, Australia) used to code and store thematic data. Data were extracted on: the phenomena of interest, methodological approach, conceptual or theoretical basis underlying the study, participant characteristics, and findings of significance to the review. Full-text screening and data extraction were carried out primarily by one researcher (WE) and checked by another (SS). Discrepancies were resolved by discussion and referral to an additional member of the review team.

### 2.4. Data Synthesis

Participant quotations and text under the headings “findings” or “results” were extracted from identified papers and entered verbatim into the JBI SUMARI software, where data was stored and coded. As our review involved bringing together two fields of literature (alcohol consumption and eating behaviour), we first coded papers which focused on alcohol use, followed by papers which focused on eating behaviours. Finally, we compared all themes recorded in order to identify those common only to both consumption behaviours. Based on Thomas and Harden’s thematic method [50] and informed by the principles of meta-ethnography [51,52], qualitative synthesis involved three core phases: line-by-line coding of findings, development of descriptive themes, and development of analytical themes. Themes and concepts identified were explored for convergent or divergent cases, using a process referred to as reciprocol translation, similar to the constant comparative techniques used in primary qualitative research. In this way, the synthesis presented here is intended to move beyond the identification of second-order constructs (interpretations offered by the original researchers) and towards the development of third-order constructs (new interpretations beyond those offered in individual studies). Key concepts and contextual details were recorded to help understand the interpretations of every paper; regular meetings were held to discuss disagreements at all stages of assessment, but most notably, to discuss the themes and subthemes that arose from the data synthesis stage.

### 2.5. Quality Assessment

Included studies were assessed for quality by two independent reviewers using the 10-item JBI Qualitative Research Checklist, available via JBI SUMARI software (SS, WE). This checklist evaluated different domains of each study including appropriateness of study design, data collection techniques and analysis methods employed. Analysed together, these 10 questions determined the quality of each paper. Although no quantifiable scoring system exists for this checklist, the researchers created one to aid the process of quality assessment. Every question was allocated a score of 1 and each paper was scored out of a maximum of 10. Studies scoring 8 or above were deemed high-quality papers; studies that scored 6 or 7 were categorised medium quality. All studies that scored 5 or less were deemed low-quality papers and subsequently, excluded from the review. There were no disagreements in the assessment of methodological quality between the two reviewers.

## 3. Results

### 3.1. Description of Included Studies

The search provided 24,327 studies for screening, of which 23,900 were excluded during title and abstract screening. A further 364 studies were excluded during full-text screening (*n* = 427, completed by WE). Ten percent of studies assessed at full-text stage (*n* = 43) were double-screened (by SS). Of these, 63 studies matched full inclusion criteria. One further study was deemed low quality and later removed after quality appraisal, leaving 62 included studies (see Figure 1). The characteristics of included studies can be found in Supplementary Table S2. Of the 62 studies included in the review, 45% (*n* = 28) of studies focused on alcohol, whereas 55% (*n* = 34) focused on eating behaviours. No identified studies focused on the interaction between alcohol consumption and eating behaviours. Fourteen papers (23%) were categorised to be medium quality, while 48 (77%) were deemed high quality. The 62 studies represented over 4188 participants aged between 11 and 17 years old. European studies dominated our review findings (*n* = 39; 63%). A further nine studies (15%) were carried out in North America; four in South America (6%); four in Australia (6%); five in Asia (8%). Only one study was carried out in Africa (2%). The study settings varied among the papers but the majority recruited adolescents from schools. The findings presented below focus only on overlapping and divergent factors which influence alcohol-use behaviour and eating behaviour for young people aged 10–17, and which might be particularly pertinent for further study amongst those for whom these behaviours co-occur. Analysis yielded five themes: (1) alcohol and unhealthy food can be used by young people to overcome personal problems; (2) young people felt that unhealthy eating and alcohol use are fun experiences; (3) young people chose food based on taste—this is not the case for alcohol; (4) exercising control and restraint over eating and drinking behaviours; and (5) alcohol and food choices can be used by young people to demonstrate identity. Themes are illustrated narratively below with representative quotes from the original studies.

### 3.2. Alcohol and Unhealthy Food Can Be Used by Young People to Overcome Personal Problems

Study participants reflected on emotional and personal issues that they wanted to overcome through drinking or eating. Young people explained how they used alcohol to forget their problems [53,54,55] or to overcome sadness and “*drown their sorrows*” [56]. One participant reflected on her drinking experience and the reasons she drank (“*Once I was drunk because I was depressed; parents were in conflict, the brother ran away from home, and I quarreled with my boyfriend...*”) [55]. Personal issues play a key role and one participant mentioned how (“*Life can go wrong and they begin to drink or to regret something. also, to forget. I think that’s the reason*”) [53]. In this way, alcohol was a cushion that can be used for comfort in tough situations as (“*…it’s a way like any other to forget or to let off steam, it depends on the person*”) [54]. Similarly, young people also used food as a way to relieve stress [57,58] and to push away negative feelings or emotions: (“*If something has happened to you, and you really want it to go away. I eat, I comfort eat, and I listen to music*”) [57]. Participants were aware of their actions and the use of food to overcome depression. Interestingly, one participant mentioned how comfort food can be used if (“*…you are depressed. It feels like the food is there for you when no one else is around*”) [57]. For this participant, these foods were replacing the human interaction he needed. Participants did not specify which type of food they ate in these circumstances, but they were concerned that (“*comfort food is probably not the healthiest stuff right, but it’s what you want to eat*”) [58].

### 3.3. Young People Felt That Eating Unhealthy Food and Drinking Alcohol Are Fun Experiences

In a number of studies, young people highlighted the joy they experienced when eating unhealthy food [59,60] and drinking alcohol [55,61,62,63,64]. Certain foods and snacks were most enjoyable (“*For each night five days in a row you probably would get Snickers, Twix, bag a chips, and a soda, and probably a night for every day that week. Or switch up on the candy, and the soda*…”) [65]. When asked which food type excited them most, participants were more excited about eating unhealthy options, such as junk food, than eating healthy food. The taste of food was an important motivating factor underlying this choice (“*Yeah. If you see a basket of fruit, you’re like “Oh. Fruit” [Spoken in monotone]. You know, we’ve had fruit forever. And then you see Cheetos^®^ and you’re like, “Wow! Food! Like, real food” [Spoken enthusiastically]. For some reason, junk food is better*”) [60]. The word “fun” was used to describe the experience of eating foods such as crisps and this was particularly the case when young people got together with friends at events such as sleepovers or parties. At these events, there was a focus on enjoyment and relaxation and unhealthy options were more appealing (“*Because [if my friends are] at a place, like at a sleepover, they don’t want to have healthy food. They want to have junk food because they just think that it’s funner [sic] to have junk food than healthy food*”) [59].

Similarly, drinking was also described as a fun experience, to have a “*good time*” [61] and to “*relax and loosen up*” [43]. A gathering or party was expected to be much more enjoyable when not sober and so some started drinking early on in the night [63]. Alcohol also allowed young people to talk with no boundaries and to be carefree around their friends [66]. Thus, they appreciated the change in behaviour that drinking offered (“*I’m not saying it’s a habit, but when you go to a party, yeah you just go there to have fun and enjoy the atmosphere. You’re free not to be yourself and to let yourself go*”) [67]. On the other hand, if alcohol was unavailable at a party, this negatively impacted young people’s attitude towards that event. Without the alcohol, the party was not the same (“*Often, many kids associate fun with alcohol in the sense that if a party is alcohol-free it’s usually a party no one attends, because if there’s no alcohol then there’s no fun. I don’t think it’s true though*”) [62]. Across studies, participants dismissed the negative consequences of drinking alcohol that might follow a heavy session of drinking. Instead, young people focused on having a good time and convinced themselves that the negative consequences would only happen to other people. One participant highlighted that when things did go wrong, young people would “*trick themselves into thinking it’s brilliant…*” [61]. For this reason, participants continued drinking anyway and described themselves as “*courageous*” for doing so [55].

### 3.4. Young People Choose Food Based on Taste—This Is Not the Case for Alcohol

Fifteen studies discussed the importance of taste when it comes to eating food [60,68,69,70,71,72,73,74,75,76,77,78,79,80]. For many young people, healthy food has an unpleasant taste (“*Healthy foods all taste awful. The manufacturers want to sell more unhealthy foods. Therefore, they add a lot of additive to make the food tasty. These foods make us feel good. The taste is exactly what we want*”) [70] with some arguing explicitly that food *“gotta taste good”* if they are to continue to eat it [71]. One participant highlighted that “*…Once I begin to eat [unhealthy food], I cannot stop*” [70] and craving ‘addictive’ foods was a regular occurrence (“*I crave the taste of soda in my mouth, especially when I see someone else drinking it*”) [60]. Further, young people acknowledged that this does not always signal hunger (“*Like the other day at lunch we were walking past Annie and she had a burrito. I wasn’t even hungry, but she had it so I was like, ‘Can I have a bite?’*”) [60], and the calorie count of unhealthy food was of secondary importance where taste was concerned (“*I don’t really care how high the calories are as long as it tastes good*”) [74]. The taste of alcohol, on the other hand, was described as bad (“*I thought that it should taste great but after I drank it, wow, it tasted horrible. I have no idea why people drink. I will not drink again*”) [66]. Across several studies, participants questioned why young people drank when the taste is so undesirable [66,81]. However, unlike the consumption of unhealthy food, where taste could be described as pleasurable, alcohol was predominantly consumed for social purposes and with the primary objective of intoxication (“*Most people drinking like that in ninth grade don’t drink because it’s good, they drink to get drunk*”) [81].

### 3.5. Exercising Control and Restraint over Alcohol and Unhealthy Food Consumption

For most participants drinking alcohol at social events was expected. Although they did it to experience fun and enjoyment, drinking alcohol was not always a good experience for them, and experienced negative consequences, which they reflected on (“*The worst feeling is being sick like and then trying to be sick and then bringing up all the beer n all or whatever you’re drinking. It’s terrible*”) [82]. Such negative events and consequences left some young people questioning why they drank and whether it was a truly enjoyable behavior (“*I think drinking’s good and then it gets worse. Like if you get completely paralytic then where’s the fun in that?*”) [82]. Many participants mentioned the desire to control their drinking habits [54,55,62,63,66]. Thus, self-control and having a “*good head*” [54] were important factors that young people considered when drinking alcohol (“*You’ve got to restrict yourself, you’ve got to have a certain amount of self-control to go to these parties. Like at a party you go to you don’t have to necessarily get drunk, you’ve just got to drink the right amount, so you have fun with your friends, you don’t throw up and you’re ok*”) [62]. Becoming uncontrollably drunk at parties was criticized as “*…stupid and just trying to show off and it’s not rational*” [62]. Nevertheless, to stop or not initiate drinking alcohol was a social challenge and very difficult to achieve. Thus, one young person highlighted that “*when I try to quit drinking, the alcohol always wins*” [55]. Therefore, to prevent alcohol from winning over their habits, narratives from young people centered on ‘knowing your limits’, achieved through experimentation and practice, almost akin to ‘learning’ how to drink alcohol (“*You know your own limitations when you drink alcohol, and my friends know their limitations too. When you are aware of your own boundaries, you know when you need to stop*”) [66]. The concept of self-control was predominantly associated with studies focusing on alcohol consumption. Just one of 34 studies focusing on eating behaviours mentioned self-control [73]. In this study, one participant spoke of the hunger associated with excess intake of sugary foods (‘‘*I see it is that when I eat something sweet, I’ll be hungry for more so it is difficult to manage, so I try to stop*”). Another participant vowed to take action and stated that “*from now on I’d like to eat fruit instead of a cake in the evening*”.

Other people also appeared to exercise control over young people’s drinking and eating behaviours. Some young people encouraged each other to drink within their limits (“*We do tend to emphasise to each other that you should know your limitations so if someone doesn’t know his limits then we would be he’s not really that cool to hang out with...I guess there is a borderline between funny and embarrassing...like the new people who come in sometimes they go over the top of it and then they know that that’s not really the way to go, so they kind of buckle down next time*”) [63]. However, peers can also be a negative influence upon behavior and some young people described being ‘coerced’ by friends to drink and gave in so as to avoid looking ‘bad’ [83]. In this particular study, much was attributed to the dynamics of the social group as drinking habits developed at a time “*when all the groups are forming...so then you want to fit in...so then you feel the pressure to do what everyone else is doing so you’re like them*” and participants feared that “*…if I didn’t drink they wouldn’t want to hang out with me anymore*”. Wanting to be accepted as part of a social group was also addressed in other studies [81,84] and participants explained that “*It’s kind of what you have to do to be a part of the group. There are lots of people experiencing such pressure. If you want to be one of the cool people, then you need to just start doing it—to try alcohol and do stuff you don’t want to then*” [81]. Young people were not always happy to consume alcohol but saying “no” to their friends was difficult to do [85]. Commonly used phrases such as “*…‘ah, go on, give it a try’…’*” [62] or “*…try it, try it*” [84] were employed by their friends to push them into trying a drink. Participants discussed being initially hesitant to try alcohol, especially if they did not intend on drinking it at a certain event. Choosing not to drink came with its own stigma and resulted in teasing and bullying *(*“*…people will start to tease you and all stuff like that, people are a lot meaner now, and if you don’t do the same what they do, they’ll start to pick on you*…”) [61]. Name-calling was another tactic used such as “*… oh you’re a chicken…*” [83] and some young people ended up drinking to please friends and avoid ridicule [86]. Some attempted to use excuses such as having to participate in sporting activities the next day and the need to remain sober, although this rarely worked to their favour (“*like if you say [to your peers] I’m not going out [and drinking] because of netball, you’ll like cop a bit of flack for that...because they [peers] don’t understand the pressure to perform...and your performance would be like totally affected [if you drank]...there’s been a few times where we’ve gone out not intending to drink and have ended up drinking*”) [86].

Pressure relating to eating habits was discussed in several studies [40,60,70,71,87,88]. One participant highlighted how “*My friends are all around me eating good [unhealthy] food and I’ll be like, “Oh I’m gonna buy that. I wanna go get one too.” Either you won’t eat or you’ll have the junk food*” [60]. Eating food that was deemed undesirable by the social group also had negative social consequences for young people including gossiping or teasing (“*If you don’t eat it while others are eating, you appear to be different from others. Others may say something [bad] behind your back*”) [70]. Eating healthy food or home-made packed food resulted in ridicule (“*[Some teenagers] just don’t eat [healthy food] in front of other people. They’re probably afraid they might get teased*”) [59]. Therefore, to avoid this, young people opted to buy food from the canteens at school, just as their friends did (“*I do not know. I believe that they might think that people will assume that their mums are preparing sandwiches for them. People feel more comfortable buying food from the canteen like everyone else, rather than having home-made food, even if it is healthier. If someone is eating a home-made sandwich, he might think that the others are going to make fun of him*”) [87]. Not only was there pressure to eat unhealthy food, but young people also felt pressured to buy expensive foods as eating cheaper foods might mean “*[peers] give you dirty looks and talk about you to your friends*” [40]. As such, participants suggested that eating cheaper brands may indicate that they are “*… poor or something. Not exactly poor but not a lot of money*” [40]. Yet, peer influence appeared to work both ways, albeit heavily dependent on the choice of friends young people kept. In other words, peers who ate more healthy food, in turn, influenced their friends to eat healthy (“*I was eating heaps of unhealthy food then and I’d see people, like, bring in heaps of fruit and then I noticed that I was eating unhealthy, so I changed to their foods*”) [88].

Perversely, overeating and drinking to excess were also frowned upon, leading to a social ‘tightrope’ that had to be navigated in order to fit in. Several studies addressed young people’s attitude to overeating [40,68,77,89,90]. Some participants sympathised with overweight and obese peers (“*I think that if you’re overweight, you still belong. I mean, you’re the same as other kids, just a little heavier*”) [68]. They argued that overweight young people may be suffering from a chronic disease and should not be discriminated against [68]. However, others had very negative attitudes towards overweight peers (“*Being overweight is not healthy. You just become so ugly. You are bullied*”) [77]. Some young people were shamed for being overweight (“*Yes, if you’re fat in school they call you ‘fatty’ and stuff like that*”) [40] and excluded from team activities at school “*so they won’t get picked on… girls think that if they’re fat, they’ll get picked on a lot more*” [90]. Further, not only were overweight young people teased for being overweight, they were also teased when they tried to adopt more healthy food choices to reduce weight, adding to the complexity of their social situation (“*I usually don’t eat at school. I mean, er’body be eatin’ stuff at lunch that ain’t really healthy, but they ain’t alot a choices. If I eat a salad or somein’ then they gonna crack on me. They say stuff like “What’s up biggie, you tryin” to lose some pounds’ and stuff. I dunno*”) [89]. Young people were also criticized for intoxication and drunken behavior [54,64,85,91,92,93]. Excess drinking was defined by one young person as “…*about you getting high, about you getting drunk, about you not being able to control yourself, about you not knowing, about you not being able to decide on your own. About you doing stupid things and being different from how you really are, also about you feeling bad the day after*” [91]. Acting out and becoming sick was not appreciated by peers, especially when at house parties (“*you don’t want them inside while they become sick*”) [64]. Young people even chose to exclude peers from events if they continued to drink excessively and were disruptive (“*We won’t invite you again if you can’t control it. You shouldn’t drink any more now*”) [93]. Some participants found drunken behaviour both “*disgusting*” while still being “*hilarious*” to watch [92]. Nevertheless, negative attitudes remained (“*Above all, in my opinion, a twelve year old girl who drinks many glasses of vodka only makes herself look ridiculous, because no one could hold that*”) [54]. Females, in particular, were criticized for being promiscuous when drunk and were looked down upon. They became a topic of conversation among their peers (“*She went skinny-dipping with another man*”) [92]. Further, it was female peers who were particularly vocal about this behavior (*“I’m not a fan of girls who get really drunk and then do things [of a sexual nature] with guys and then blame it on the fact that they were drinking...I really don’t like that because I kinda go, well I’d never get to that point*”) [86].

Such pressure on eating behavior and alcohol use often led to young people conforming to fit in with others (“*I simply go with the flow of my era and I do not confine myself to eating beans, for instance. That is a ‘no-no’ for our times. I am a follower of today’s fashions. Young people today believe in eating in Pizza Hut, hamburgers,* etc. *[pause] this shows that I am ‘in’ and not cut off from the rest*”) [87]. In relation to alcohol, they tended to drink in the same ways in order to reach the same consumption level (“*It is so much more fun when all of us are on the same level—when there isn’t anyone higher or lower than the rest of us*”) [94]. This was not something they always wanted to partake in, however, they choose to do it regardless of whether they liked the experience or not (“*It happened to me as well, we were at the beach, we ate there, I don’t like beer, but everyone drank and then I took the beer and drank it all and bleah*”) [54]. Those who did not drink were excluded from social events with their peers and were described as “*weakling*” or “*a loser*” [55], placing immense social pressure on adolescents to participate in drinking sessions as they did not want to “*…just stand there looking*” while everyone else drank alcohol [84]. For many this was “*… a bit embarrassing*” [66]. Instead, the social norm for adolescents was to drink and be “*…caught up in the spur of the moment. You see all your pals drinking and…then you end up just joining in with them*” [61].

### 3.6. Alcohol and Food Choices Can Be Used by Young People to Demonstrate Identity

Drinking alcohol allowed young people to demonstrate a sense of identity, be perceived as cool, and to be praised for it (“*…your friends think you’re great, and they’re good if you drink…*”) [55]. Part of maintaining a “*cool*” image was to show off in front of friends and “*brag*” about drinking while being underage [95]. This helped them appear tough in front of their friends (“*They want to make themselves look hard, like oh yeah I drink*”) [83]. Young people tended to exaggerate their drinking experience and this in itself was perceived negatively by their peers (“*They’re loads of people who come in the next day, they exaggerate so much and they’d be like, ‘Oh I drank two bottles of Buckfast and two wee bottles of vodka’ and all this shit like you would be dead*”) [95]. Coolness, popularity and being trendy were interlinked (“*It’s because it’s the fashion. It’s the fashion that we get drunk every weekend (laughter)!*”) [84]. Not everyone shared this sentiment and one participant had a different view (“*Young people only drink because it’s supposed to be cool. They are trying to act tough in front of groups of guys or girls. That they kind of “yes, we are drinking and we are really cool*”*. That was how it was earlier but now, at least in our grade and maybe ninth grade as well, they have started to think that it is really childish to stand there trying to be tough by drinking*”) [81]. Nevertheless, the majority of young people who drank remained popular and influential among their peers (“*If one starts who may be popular and others, like, fancy him and think that he’s cool. And if he starts, like, to drink, then he might influence a lot of other people. Because they want to be in his group*”) [91].

Being ‘cool’ and demonstrating a sense of identity was also an important factor when choosing which foods to eat around their peers [40,69,87]. Certain foods such as sweets or fast food (for example, hamburgers or pizzas) were viewed as trendy (“*YES, YES the Mac style is more modern. It is more in, modern for young people*”) [87]. Young people felt pressure to “*be cool and be like everybody else—It is just a chain reaction*” [69]. Subsequently, those who chose other food options such as home-made and healthy options were ostracized (“*The one who eats healthy food will be more of a dumb person…[He] cannot be modern or cool*”) [87]. This extended beyond simply eating certain foods and included how food products looked or were branded, and how they could be eaten (“*You wouldn’t have a yoghurt I don’t think because then you’d have to have a spoon!” “Yes.” “That is not cool.” Moderator: “Spoons are not cool?” “Not.” Moderator: “Really?” (All laughing) “You look stupid getting a big metal spoon out of your bag.*”) [40]. One participant highlighted that part of the appeal of eating certain unhealthy food products was the ability and ease of sharing it with friends. This built social bonds and cemented popularity among peers (“*But people are more excited when somebody brings sweets—you are more popular. It is easier to say do you want to have a piece of chocolate than do you want a bite of my apple*”) [69]. Media and advertisements played an influential role in choices of food and alcohol brands. Young people highlighted the role of advertising and social media in promoting certain alcohol brands [63,79,86,96]. Alcohol promotion lingered in the memories of participants (“*Ad[vertisement]s for Smirnoff are really classy and you really want to drink them ‘cause they look awesome. Because everyone’s doing it [drinking Smirnoff] you want to do it too*”) [86]. Participants were curious to discover the taste of the drinks that were promoted in advertisements and certain brands conjured up a persona that young people wished to imitate (“*I seen one of the adverts off Jack Daniels...I was like ‘oh I wonder if I’d do that if I’m on Jackie D’s’ and I just had to get a bottle of Jackie D’s and nowt happened...I liked the taste of it and that. Same with Southern Comfort. I’ve seen the advert for that and I was like ‘oh’ and decided to have a drink of it*”) [63]. While advertisements promoted both healthy and unhealthy food, those that focused on unhealthy food were perceived as more exciting and fun (“*The advertising promotion of unhealthy food is very good. For example, a candy is able to change the color of your tongue. There are always some new foods coming up. Young people will find them playful*”) [70]. Like the alcohol industry, the food industry also focused on the fun aspects of eating unhealthy foods. The lifestyle associated with this was more appealing to young people (“*Well, they advertise it a lot more than fruit. Like have you seen the swirly commercial? The kid comes home and his hair’s all crazy because he goes on a roller coaster. And he is the guy that makes it all fun. You know, it’s like we’re gonna go on a trip and eat these Cheetos^®^*”) [60].

In many studies, young people wanted to be viewed as mature individuals (“*We started to party in the seventh grade and I have always felt, how should I put it, a bit ahead of the others…a bit older…Some of the others have only just started to party now*”) [97]. They wanted to drink alcohol to mimic adults and be viewed as more mature individuals by their peers (“*Drinking is associated with being an adult, like you know, smoking, doing all those things which like adults do, if you are younger and you do the things adults do then it makes you seem older and I think that’s what they want*”) [83]. This helped young people to “*feel superior to others. You feel great!*” [84]. Boasting about drinking experiences helped elevate this feeling and participants used phrases such as “*I got totally smashed*” [84] to help them feel older amongst their peers. Part of appearing older was choosing different types of alcohol beverages (“*when I get older I’ll start drinking beer and wine...I think they’re classy drinks like they’re drinks you’ll have at a restaurant rather than the one that like you drink at a party*”) [86]. On the other hand, unhealthy eating habits were associated with being young and modern; such young people were viewed as attractive and independent (“*When you have delivery food you show a different character, more outgoing and attractive than having home-made food prepared by your mum or grandmother. You come over as a person with a degree of independence who does not depend that much on his family; you look cooler*”) [87]. Healthy foods were eaten by more ‘mature’ young people and, unlike alcohol use, this was an undesirable trait to have (“*I think, it’s more mature to say that you want a piece of fruit—and then if somebody asks if you want to go downtown for a bag of crisps, that’s what you’ll do*”) [69].

Ultimately, young people wanted to “*feel like being grown up and to be the coolest person in school*” [91]. Achieving this balance was complex, and participants believed that they would change their drinking and eating habits as they grew older (“*When I’m older, I’ll probably still drink, but not to get drunk, just to enjoy it, whereas at the moment I’m probably just drinking to get drunk*”) [83]. Thus, despite knowing the negative health risks of excessive alcohol intake or unhealthy eating behaviours, participants did not appear concerned. For alcohol, young people were concerned about more immediate consequences such as having a hangover or being caught by the police for a misdemeanor (“*…young people think about the short term more than the long term because they think about what they’re going to look like right now, than think about what will happen when they’re older.*”) [61]. The role alcohol played in bodily harm or long-term diseases were of secondary importance to young people, with these findings mirrored in studies looking at unhealthy eating habits [87,98]. Instead, body image was a greater and more immediate concern than future health risks (“*We probably should be thinking about our health, but personally I think more about my body…If I eat something that’s unhealthy I think, Oh no! That will make “me fatter”. I don’t think about my future health*”) [98]. Peer influence also appeared to have a role to play. One participant described that her peers may dismiss health concerns related to unhealthy eating habits. They would even tease more health-conscious young people who choose to eat more nutritious foods (“*…you are afraid that something will happen to you, so they might make fun of you that you are afraid and you worry about your health. They might say that: ‘We are young and we do not care about all these things’*”) [87]. Further, young people expected to change their eating and drinking habits as they got older. For example, they differentiated between teenage drinking and adult drinking. While they assumed that young people drank to get drunk, they did not expect this behaviour from adults as ‘*…adults are more in control than young people*’ [81]. Adult drinking was associated with meals and family gatherings and participants believed that, unlike their own behaviours, the “*[adults are] done running around partying and drinking*” [81]. One participant argued that this was because “*…when you grow up you become smarter…*” [62].

## 4. Discussion

This review and qualitative synthesis demonstrated that there are a number of socio-cultural, interpersonal and structural influences upon young people’s (aged 10–17) unhealthy eating behaviours or alcohol use which cut across *both* behaviours. Studies included in this review suggest that young people regularly feel pressure to drink alcohol and eat unhealthy food, particularly when around their peers. There appear to be clear social consequences and image concerns when it comes to the restriction of either of these behaviours, leading to navigation of what could be described as a social ‘tightrope’. In other words, young people must fit in but not go overboard. In relation to alcohol use, those who do not drink as well as those who drink too much can lose favour with peers. This paradox has previously been described among young adults as ‘calculated hedonism’ [99]. Similarly, those who eat too healthily, as well as those who over-indulge, and become overweight, can be subject to ridicule, suggesting that young people must consume in very specific, rigid ways in order to be accepted. For many, this may be an impossible feat, with clear impacts on young people’s physical and mental health demonstrated in wider literature [100,101]. Such pressure to behave according to narrow, pre-defined categories appears to be reinforced by wider structural mechanisms, including but not restricted to, social media use [102] and commercial drivers, such as product marketing [36,103]. Previous work suggests that marketers (‘big alcohol’ and ‘big food’) reinforce aspects of the social ecology by encouraging links between alcohol, food and aspects of identity, culture and personal reward [63,104]. Indeed, this relationship is well underway in some young people by mid-adolescence [105]. However, this is an iterative rather than linear relationship. In other words, industry feeds off young people’s concerns, as well as leading them, meaning that it may be difficult to disentangle the ‘real’ concerns of young people (‘knowing your limits’) from those seeded by industry through marketing and ‘educational’ programmes, including alcohol programmes which are run in UK schools, and affiliated with industry (such as ‘Drinkaware for Education’). Further, commercial determinants have seen expansion in recent years into new directions such as energy drinks and gambling [106,107,108], and warrant further exploration in future research. Such exploration should examine the holistic impact of commercial determinants upon young people’s social, emotional and cultural worlds, rather than investigating these drivers in isolation.

The volume of studies identified by this review means that we have reported only what we concluded to be major themes. We also reported only themes which cut across both eating behaviours and alcohol use. This is not to say that there were no other influences on young people’s alcohol use and eating behaviours. For example, parents and extended family members appeared to exert an influence on both behaviours within a smaller number of papers. Young people discussed the importance of a trusting relationship with their parents, the ability to have open discussions about both alcohol use and nutrition and the role of both parental disapproval and initiation when it comes to their child’s alcohol use. As anticipated, we found no literature focusing on how young people consume food and alcohol simultaneously. Yet, one way in which to tackle a growth in obesogenic and alcohol-related harm may be to examine the overlapping influences in these behaviours at the point in which they initiate and accelerate (late childhood/early adulthood) and use this knowledge in the design of interventions which link rather than separate out food and alcohol consumption. Emergent research with young adults suggests eating patterns linked to alcohol use are not tied only to hunger but to sociability, traditions and aspects of identity. Further, young adults conceptualized and calculated risks to their weight, appearance and social status rather than to their long-term health (Scott et al., 2019, in press); whilst a raft of quantitative studies have identified that energy intake from alcohol, type of beverage and drinking pattern are associated with excess body weight and weight gain amongst young adults [21,26]. However, these attitudes and behaviours may well be established in earlier years, suggesting the need to conduct work examining the detailed links between alcohol use and eating behaviour with younger age groups. Finally, young people felt that their alcohol use and food choices would change as they got older, and tended to ‘discount’ potential negative consequences. Again, this argument is well supported in both fields of literature [109]. Narratives of delay discounting do not appear to be restricted to adolescence and have been demonstrated to track into young adulthood [28]. For example, young people in this review acknowledged that the taste of food was important, whereas the taste of alcohol was not. However, young people suggested that the taste of alcohol would be more meaningful to them when they were older.

There are several limitations of our review which should be acknowledged. First, we defined ‘young people’ to be aged 10–17. We thought very carefully about this definition. Whilst in some developed countries the legal drinking age can be higher (aged 21 in USA), we were informed by the UK legal drinking age of 18. Further, turning 18 has further connotations in young people’s lives. For example, turning 18 can signal transition from children to adult health services, transitions from further education to higher education and can mark major life transitions, meaning that the environments of 16 and 17 year-olds can be experientially different to that of those aged 18 and over. Second, papers focusing on both addiction and weight control behaviours and syndromes (such as bulimia and anorexia) were deliberately excluded from our review. We felt that papers in this field focused on targeted, psychological disorders, whereas our review synthesized literature on wider cultural, inter-personal and social perspectives on eating and drinking behaviours in adolescence. Third, many of the papers in this review did not explicitly reflect upon differences between socio-economic groups. Yet, recent work suggests that socio-spatial patterning of outlets selling potentially health-damaging products such as fast food, alcohol and tobacco tend to cluster in deprived areas [110]. Further, obesity and alcohol-related mortality and morbidity are high in socioeconomically disadvantaged populations compared with individuals from advantaged areas, characterized in terms of occupation, income or educational attainment [111]. Fourth, due to time and resource restrictions, we did not double-screen all identified studies at full-text stage. Rather than employ single screening, we took the decision to double-screen 10% of full-text studies identified in order to add rigour to our review findings. Further, whilst not all results were formally double screened, the author with responsibility for full-text screening (WE) discussed any uncertainties and discrepancies across all 427 full-text papers assessed with the second reviewer (and lead author, SS) at all times. Fifth, we attempted to contact authors whose papers we were unable to obtain during database searches, with very little success. Finally, we did not analyse the data according to country of origin and, therefore, cannot make assumptions based on geographical or cultural traditions or differences.

Findings from this study have important implications for intervention development, as well as UK public health policy and practice. Themes identified in this review, and in recent parallel work conducted by the lead author with young adults, will be used to inform a qualitative exploration of young people’s (aged 10–17) views regarding their eating behaviours and alcohol use. Combined, review and interview data will be used to develop a model structure, or logic model, of the influences upon both unhealthy eating behaviours and alcohol use amongst young people. It is anticipated that this model structure will be used in future participatory design work with young people, which aims to generate ideas for a dual-focused intervention to reduce alcohol consumption and excess body weight among young people, and to promote a positive approach to body image, physical and mental health.

## Supplementary Materials

The following are available online at https://www.mdpi.com/2072-6643/11/8/1914/s1, Table S1: Review Concepts and Associated Search Terms, Table S2: Characteristics of Included Studies.
