# Supplementary material for: Socio-Ecological Influences on Adolescent (Aged 10–17) Alcohol Use and Unhealthy Eating Behaviours: A Systematic Review and Synthesis of Qualitative Studies
Source: Nutrients. 2019 Aug 15;11(8):1914. doi: 10.3390/nu11081914 (PMC6722644; doi:10.3390/nu11081914)
Supplement: Supplementary file 1 [file nutrients-11-01914-s001.pdf]

Supplementary Table S1: Review concepts and associated search terms

SPIDER concept	Search Terms
S - Sample: Young people	Child OR children OR adolescent OR youth OR young people OR young person OR young adult OR kid OR teenager OR under-age OR student
PI - Phenomenon of interest: alcohol consumption OR unhealthy eating behaviours	Alcohol drinking OR alcoholic beverages OR alcoholic intoxication OR alcohol consumption OR alcohol misuse OR alcohol abuse OR alcohol use OR risky drinking OR excessive drinking OR problem drinking OR binge drinking OR hazardous drinking OR unsafe drinking OR unhealthy drinking OR drunk OR eating behaviour OR unhealthy eating OR unhealthy diet OR food choice OR food preferences OR food habits OR food intake OR feeding behaviour OR energy intake OR fast foods OR carbonated beverages OR obesity OR overweight OR overnutrition OR overeat OR over-eat OR excess weight OR body size OR body mass OR body weight OR diet OR nutrition OR underweight OR undereat OR under-eat OR undernutrition OR fruit OR vegetable OR portion OR serving OR junk food OR fast food OR processed food OR calorie-dense OR calories OR convenience food OR dietary fat OR dietary sugar OR dietary salt OR fizzy OR sugary OR snack OR takeaway OR takeout OR carry-out OR frozen OR ready-meal
D - Design: qualitative research	Interview OR grounded theory OR ethnography OR interpretative phenomenological analysis OR phenomenology OR focus group OR content analysis OR thematic analysis OR constant comparative OR participant observation OR narratives OR field notes
E - Evaluation: Experience	perceive OR perception OR perspective OR view OR experience OR attitude OR belief OR opinion OR feel OR know OR understand
R - Research type: Qualitative and mixed methods	Qualitative OR qualitative analysis OR qualitative research OR mixed methods

Supplementary Table S2: Characteristics of Included Studies

Study:	Data collection / analysis methods:	Theoretical approach outlined:	Country / Setting:	Phenomena of interest / study aims:	Participant characteristics / sample size	Key findings:
**Acier et al., 2015**<br><br>**Alcohol**	Interviews and focus groups	Yes – phenomenological inquiry	France<br><br>Middle or secondary school and recreation centres in the Pays de la Loire region	To examine consumption behaviour according to the meaning assigned to it by the participants and according to the way adolescents spoke about their consumption.	Interviews: n=34 (13 boys and 21 girls), aged 12-17.<br><br>Six focus groups conducted, including 23 adolescents (19 boys and 4 girls), aged 12-17.	Highlighted the influence of families on initiating drinking and of peers on current consumption.<br><br>Adolescents were aware of the negative consequences that alcohol use might entail.
**Ander et al., 2015**<br><br>**Alcohol**	Ethnographic approach; data collected via interviews.	No explicit theoretical approach outlined, however the authors describe a sociological approach to the concepts of space and place.	Sweden<br><br>Small town	Explores the significance of arenas of underage adolescent binge drinking in some Swedish small towns and the meanings these have in terms of place and space for young people involved.	N=28 adolescents aged 16-17: 15 boys and 13 girls	Key restricting factors to binge drinking were lack of home arenas, lack of money and lack of the ”right” group of friends.<br><br>The level of binge drinking was also lower in homes where parents were present in another section of the home or were out for the evening.

**Ashcraft et al., 2013**<br><br>**Food**	In-depth, semi-structured interviews to obtain descriptions of the lived experiences of adolescents within the themes of self, home, school, and society.	Yes – phenomenological inquiry	USA<br><br>Participants were recruited from an inner-city obesity clinic located in Memphis, Tennessee.	To: (1) examine how inner-city African-American adolescent males experience and manage their obesity, and (2) investigate the core components of inner-city African-American adolescent males' explanatory models of obesity.	N=13. Participants were: (a) male (b) of African-American descent, (c) between the ages of 13 and 17 years, (d) diagnosed with obesity as indicated by a BMI in the >95th percentile (e) English speaking.	A conflict exists between the themes of self and school and the themes of home and peripheral relationships.<br><br>The themes of self and school elicited generally negative associations, while home and peripheral relationships elicited generally positive associations. It was within the home that adolescents described feelings of comfort and acceptance.
**Bakken et al., 2017**<br><br>**Alcohol**	Interviews and focus groups.	Yes – sociological approach to symbolic boundaries and identity.	Norway<br><br>Three schools from the South-East region of Norway (two urban and one rural), two from a larger city in Western Norway, and two schools	To explore: (i) how young adolescents describe the difference between adult and youthful drinking, and (ii) how is this distinction used in social identity and symbolic	29 focus groups and 32 interviews with 141 12–13- year-olds (71 boys and 70 girls).	Participants in this study believed that adolescents that drank alcohol were portrayed as childish, stupid, and subject to peer pressure.

			from Northern Norway (one urban and one rural).	boundary work among young adolescents?		Young adolescents were critical of alcohol in general, but clearly distinguished between adolescent and adult drinking.
**Banna et al., 2016**<br><br>**Food**	Semi-structured interviews and pile sort activities.	Authors describe using a previously developed theoretical framework which they do not name: individual (intrapersonal), Social environmental (interpersonal), physical environmental (community settings), and macrosystem (societal).	Canto Grande, a peri urban shantytown community in San Juan de Lurigancho Lima, Peru.	To gain insight into socio-cultural influences on eating in adolescence.	Adolescents 15–17 years of age (n=14).	Family was consistently described as having a positive influence on food habits and skill. Potential peer influences on eating habits were also identified.
**Bech-Larsen et al., 2010**<br><br>**Food**	Focus groups	No theoretical perspective explicitly outlined.	Copenhagen, Denmark	To explore adolescent perceptions of snacking conventions and dilemmas.	Omnivorous, generally healthy, ethnic Danish adolescents (13-15 years of age) and living with their parent(s) (not clear how many participants took part)	The study identified two forms of adolescent snacking: ''in-between meals'' and ''fun snacks''. In-betweens are primarily purchased by parents and are

						thus subject to parents' preferences for healthy food.<br><br>However, usually snacks are purchased by young people themselves.<br><br>Snacks are often the focal point of social interaction.
**Chan et al., 2009**<br><br>**Food**	Focus groups	Yes - Interpretivist approach	Hong Kong<br><br>Schools	To explore Chinese adolescents' perceptions of healthy eating, their perceptions of various socializing agents shaping their eating habits, and their opinions about various regulatory measures which might be imposed to encourage healthy eating.	22 eighth and ninth grade adolescents (aged 13 to 15) mixed by gender, and local versus international school students.	Participants perceived a balanced diet and regular meal times as the most important attributes of healthy eating. Participants were most likely to eat unhealthy food at parties, during festivals, and when socializing. They reported that mothers and teachers often advise them to eat healthy foods.

**Chan et al., 2016**<br><br>**Food**	Focus groups	Yes - Interpretivist approach	Junior high school in Mainland, China	To explore snacking behaviour and perspectives on healthy and unhealthy food choices among adolescents.	24 Chinese adolescents aged 12 to 13 years studying in Grade 1 of a public junior high school	Adolescents consumed both healthy and unhealthy snacks, eating large amounts of fruit after dinner.<br><br>Taste and imagery were important in food choices for adolescents. They preferred the artificial flavour of unhealthy food to the mild flavour of healthy food.
**Christiansen et al., 2013**<br><br>**Food**	Interviews, participant observation and focus groups	Yes - combined a social ecological model and social cognitive theory.	USA<br><br>Participants were recruited from Baltimore City recreation centres	To study the environmental factors influencing the food-related habits of low-income urban African American adolescents	Participants were African American and aged 10–16 years.<br><br>20 adolescents took part over the course of 18 in-depth interviews and 2 focus group discussions	Six thematic categories emerged and were organized into 4 environmental contexts: the neighbourhood (accessibility of food and safety of neighbourhood), the school (school food environment), the family (family health history, role modelling, and monitoring)

						and peer (peer behaviours).
**Dapi et al., 2007**<br><br>**Food**	In-depth interviews	No theoretical perspective explicitly outlined.	Schools in Yaoundé urban and Bandja rural areas of Cameroon.	To investigate factors influencing rural and urban adolescents' food perceptions during a time of nutritional transition in Cameroon, Africa	Fifteen adolescents aged 12-15 years old.	Factors influencing adolescents' food perceptions differed from urban to rural areas.
**Davies et al., 2013**<br><br>**Alcohol**	Focus groups	Yes – prototype willingness model (PWM)	England<br><br>A single secondary school.	Explored the prototype willingness model constructs among young people in England and the nature of any alcohol prototypes held by the young people that potentially could be targeted in an intervention.	13 males and 14 females aged 11–13 and 16–17.	Drinkers were often described as 'cool' and sociable people who liked to have fun. They intended to drink and usually to get drunk when they went out. The influence of peer pressure was noted.
**Demant and Jarvinen, 2006**<br><br>**Alcohol**	Focus groups	Yes – symbolic interactionism, Erwin Goffman's dramaturgical approach to social interaction and the post-structuralist reasoning of Judith Butler.	Denmark<br><br>Small town	To demonstrate how the struggle for social recognition – with alcohol as the central marker – transpires in groups of teenagers in Denmark	28 focus groups.<br><br>Girls aged 14 and 15 years old, from 8th-form students.	Teenagers who do not drink are constructed as lacking a ''stable identity''.<br><br>Girls who drink are seen as being more ''mature''.

**Demant and Ostergaard, 2007**<br><br>**Alcohol**	Focus groups	Yes – suggests a new theoretical approach which combines structural positioning of alcohol use with phenomenological inquiry.	Denmark<br><br>Three lower secondary schools	What is the meaning of partying from the perspective of adolescents' leisure life-world, and how does alcohol consumption fit into the phenomena of partying?	37 focus groups conducted.<br><br>15-16-year-old adolescents in the eighth, ninth and tenth grade.	Drinking is associated with partying. These parties bring young people together. Friendship is a central aspect of the party.
**Dodson et al., 2009**<br><br>**Food**	In-depth interviews.	Yes – social cognitive theory.	USA<br><br>Inner-city neighbourhood in East Baltimore	What are the dietary habits of African American adolescents in East Baltimore? What is the influence of the family, school and neighbourhood environments on food choices among African American adolescents? How do African American adolescents conceptualize a "healthy" diet?	15 interviews with 9 adolescents aged 12-17.	Family structure, economic resources and past experiences influenced what food meant to adolescents.<br><br>Healthy food in school and local stores is limited.<br><br>Terminology to categorize foods was identified, including the term "home foods".
**Ensaff et al., 2015**<br><br>**Food**	Focus groups	Yes – grounded theory	UK<br><br>Large inner-city secondary school in Yorkshire, with	Adolescents' attitudes and perceptions towards plant-based foods, from their	Adolescents aged 14-15 (n=29)	There is peer influence upon food choices.<br><br>Study findings outlined the

			higher than average Free School Meal (FSM) profile	perspective and within the context of their food choice parameters		importance of convenience and price when it came to selecting food.<br><br>Young people were generally reluctant to try new foods.
**van Exel et al., 2006**<br><br>**Food**	Discourse analysis using Q-methodology	No theoretical perspective explicitly outlined.	Netherlands<br><br>Schools in the cities of Amsterdam and Nijmegen, and a summer camp for obese youths.	To uncover young people's discourses about their health lifestyle, focusing on overweightness.	Total of 113 adolescents aged 12-15; three from the summer camp group (two girls, one boy); 110 pupils from the two secondary schools.	Young people seemed uninterested in their future health, but for different reasons across different discourses: (1) because they feel physically fit (''carefree sporty''); (2) are generally satisfied and happy (''contended independent''), or (3) because they simply do not care (''indifferent solitary'').
**Fraga et al., 2011**<br><br>**Alcohol**	Semi-structured interviews	Yes - Interpretivist approach	Portugal<br><br>Adolescents from public and private schools	Frequency and determinants of alcohol use among 13-year olds	30 adolescents (15 girls and 15 boys)	Adolescents felt that people drink alcohol because they feel upset and it is a way to forget problems.

						People also drink alcohol for social motives, such as being at parties and with friends.<br><br>Adolescents perceived that drinking consequences only affect the drinker him/ herself and not others.
**Frederiksen et al., 2012**<br><br>**Alcohol**	Semi-structured interviews	No theoretical perspective explicitly outlined.	Denmark<br><br>High School, Copenhagen	Alcohol use in the social setting of parties, consumption strategies, the parents' role, and how adolescents view the health consequences of drinking.	Five girls and five boys, all ethnic Danish students, 16–17 years old and moderate drinkers.	Adolescents argued that it was important to drink at parties if you want to feel socially included. They wanted to be on the same level of intoxication as others. Parents played a minor role in relation to the respondents' alcohol behaviour.
**Gavaravarapu et al., 2015**<br><br>**Food**	Focus groups	Yes - oriented to the popular medical narrative of obesity	India<br><br>Randomly chosen schools catering to	To assess the differences in risk perceptions of overweight/obes	Seventy-nine adolescents (ages 11–14 years).<br><br>10 focus group discussions (5 each with overweight/ obese and normal-weight groups)	Young people preferred using the media to learn about nutrition and

		and the RPA framework of health communication, which explores the interactive effects of perceived risk and perceived self-efficacy to identify differences in information seeking among 4 attitude groups (responsive, avoidance, proactive, indifference).	middle-income groups from 5 geographical zones of the South Indian city of Hyderabad	e and normal-weight adolescents about obesity and associated risk factors.		discussed the importance of physical activity.
**Harrison and Jackson, 2009**<br><br>**Food**	Interviews	Yes – social constructivism	Canada<br><br>three public schools in Halifax, Nova Scotia.	Symbolic meanings that young people associate with food.	Aged 13-15 (seven girls, six boys).	Participants classified foods into healthy and unhealthy groups, as well as into an "in-between" group that included nutritionally enhanced foods.<br><br>Healthy and unhealthy foods were linked to a variety of physical, social,

						and emotional meanings.
**Harvey and McKay, 2017**<br><br>**Alcohol**	Focus groups	Yes – consideration of future consequences (CFC).	UK<br><br>16 high schools in Northern Ireland and Scotland	Perspectives on adolescent alcohol use and consideration of future consequences (CFC)	Aged 13–14; 129 pupils participated (male = 62, female = 67), with an average of eight participants per group.	Majority of adolescents do not consider the consequences of alcohol consumption at all.<br><br>Young people experienced peer pressure to drink alcohol and to fit in with friends/ consume alcohol because they knew that they do not need to deal with long-term consequences immediately.
**He et al., 2012**<br><br>**Food**	Focus groups using qualitative content framework analysis	Yes – Theory of Planned Behaviour.	Denmark<br><br>Schools serving either Organic and non-organic foods to adolescents	To investigate whether organic school meals can be an effective strategy to provide healthy food to children and promote their healthy eating habits	24 pupils from organic schools; 25 pupils from non-organic schools	In non-organic schools, some pupils expressed that they preferred not to eat school meals because they feel that the food is too 'serious'.<br><br>Pupils in organic schools were more

						inclined to purchase food from the canteen.
**Hjelkrem et al., 2013**<br><br>**Food**	Focus groups	No theoretical perspective explicitly outlined.	Norway<br><br>4 different schools in Oslo and 2 other municipalities in Norway	What adolescent girls mean when they talk about healthiness and slimming through weight-control behaviour.	28 girls in sixth grade (11 years old) and 24 girls in tenth grade (15 years old).	Motivation for healthy living was to ''keep the body in good shape,'' and ''feel good about yourself.' To keep slim girls suggested to stay away from typically fat or sugar-rich food such as potato chips, junk food, candy, and chocolate.
**Hunsberger et al., 2015**<br><br>**Food**	Mixed-method design<br><br>Interviews	No theoretical perspective explicitly outlined.	USA<br><br>Jefferson County Middle School (JCMS), Madras, Oregon.	Investigate the impact of calorie posting at point-of-purchase in a middle school cafeteria	Students aged 11-15 years in 6th–8th grade.<br><br>N=32	Students were aware of the adolescent obesity epidemic and thought it was the school's responsibility to help stop this trend and aid the students in achieving a healthy weight.<br><br>Nutritional knowledge was related to home environment.

						Taste was declared the most important factor; calorie labels helped young people to make healthier choices but not always.
**Ioannou, 2009**<br><br>**Food**	Interviews	Yes – grounded theory	Urban area of Nicosia in Cyprus	Experiences relating to the choice of fast and/or healthy foods/ to explore how 'fast' foods and 'healthy' preferences have a lifestyle focus.	25 young people (12 male, 13 female) aged 15-17.	Fast foods were regularly defined by participants as a sign of a youthful image.<br><br>Healthy food choices were viewed as being in opposition to the normal image of being young. Therefore 'fast' and 'healthy' eating choices have symbolic values.<br><br>Participants did interpret the division between fast and healthy foods in terms of health consciousness, but in many

						cases consciously chose to not be mindful of health because it was irrelevant to a 'proper' youthful image.
**Järvinen and Østergaard, 2009**<br><br>**Alcohol**	Focus groups and interviews	Yes – inspired by the work of Beck and Giddens on individualisation theory and reasoning on governmentality.	Denmark<br><br>Upper secondary schools, one school in Copenhagen and two schools in smaller towns.	Relationship between the drinking habits of Danish adolescents and the upbringing ideals and alcohol rules of their parents.	28 focus groups; 14 interviews, interviews were repeated one year later with the same adolescents.<br><br>In total, 117 young people (63 girls and 54 boys) aged 14-16 participated in the focus groups.	Young people who drink very actively, and whose parents do not intervene in their drinking, present themselves as being "ahead" of their peers and no longer in need of their parents' monitoring.<br><br>Parents who do not let go of their adolescents are seen as a hindrance to social development. Not being allowed to drink alcohol affected interactions with peers.

**Johnson, 2013**<br><br>**Alcohol**	Focus groups	Yes – Goffman and performativity.	Northern Ireland<br><br>12 schools in Belfast that reflected the gender and class composition of the area	Performance authenticity: to test if peers deem that the drinking performances of their fellows are appropriate, genuine and valid representations of an individual's wider social profile	Aged 14-15 (n=251)	'Acting hard' was associated with a 'badass', 'cool', streetwise, tough identification that made a teenager socially 'older' than other young people.
**Johnson, 2011**<br><br>**Alcohol**	Focus groups	Yes – influenced by the 'New Sociology of Childhood' which prioritises the voice of the child.	Northern Ireland<br><br>Three schools in East Belfast	To challenge the discourse that underage drinking is inevitably 'deviant'	40 teenagers aged 14–15 years. Teenagers were from working-class backgrounds.	Respondents associated alcohol with socialising and having fun.<br><br>Drinking aided the negotiation of friendships, provided a convenient excuse for disinhibition and augmented confidence in social and sexual situations.
**Jonsson et al., 2017**<br><br>**Food**	Focus groups	Yes – interpretivist approach	Sweden<br><br>Low SES multi-cultural community.	To study the factors that undermine the healthy habits of adolescents	10 focus groups.<br><br>Total sample of 53 adolescents aged 12-13.<br><br>Males = 21<br>Females = 32	When sad or depressed, respondents tend to eat more sweet and high-fat foods.

						Young people were dissatisfied with the lack of variety and options in the school cafeteria and with the availability of unhealthy foods.
**Jørgensen et al., 2006**<br><br>**Alcohol**	Semi structured interviews and participant observations	No theoretical perspective explicitly outlined.	Denmark<br><br>Rural	To study the strategies of harm minimization employed by teenage drinkers	N=32 (13 boys, 19 girls) aged 15–16.	Young people worried more about social than health risks.<br><br>Young people monitored their own level of drunkenness and preferred drinking in the company of well-known and trusted peers. They supervised one another throughout drinking episodes and intervened in each other's' drinking / used personal experiences in the planning of future drinking episodes.

**Katainen and Rolando, 2015**<br><br>**Alcohol**	Focus groups	Yes – guided by Reception Analytic Group Interview Technique (RAGI).	Italy and Finland<br><br>Schools	To study the role of loss of control in adolescents' drinking and the ways it contradicts and echoes the ideals of self-governance	28 focus groups (18 groups conducted in Finland and 10 in Italy)<br><br>148 adolescents aged 15 years. In Finland, participants were in 9th grade, in Italy they were in the 2nd year of high school.	Social pressure was a common feature behind binge drinking.<br><br>Young people related binge drinking to negative emotional states and interpreted visible drunkenness as an indicator of underlying problems.<br><br>Vomiting is associated with lacking experience with alcohol.
**Katainen et al., 2015**<br><br>**Alcohol**	Group interviews	Yes – guided by Reception Analytic Group Interview Technique (RAGI).	Finland<br><br>Schools in Helsinki	To demonstrate how adolescents make sense and articulate the risky elements of different kinds of drinking situations	18 group interviews.<br><br>Finnish ninth graders aged 14–15. A total of 89 students were interviewed, 49 girls and 40 boys.	Young people were appalled by the idea of drinking alone: it was thought that drinking alone might be explained by loneliness or depression.<br><br>It is expected that the group will always look after a friend who is in a more

						vulnerable state.
**Knox et al., 2016**<br><br>**Alcohol**	Reflective writing task	Yes – guided by narrative approaches, consumer socialisation and social learning theory.	Australia<br><br>Schools	To study how adolescents perceive and experience alcohol, through elicitation and analysis of alcohol narratives.<br><br>To assess the extent that students critically analysed alcohol experiences.	1,214 adolescents were invited to write a story about an experience that involved alcohol.<br><br>The mean age of the group who participated in the program was 15.1 years with approximately equal numbers of males (n=746, 51.4 per cent) and females.	Learning situations were predominantly parties and social gatherings.
**Kolind, 2010**<br><br>**Alcohol**	Focus groups	Yes – Bourdieu's approach to social class.	Denmark<br><br>Students from three 9th grade classes of a provincial Danish secondary school	To describe and analyse differences in the drinking and partying practices of young Danes, and to show how these can be understood in the light of theories of social class.	24 focus groups<br><br>66 adolescents aged 14-16.	Mainstream Youngsters: Adolescents reproduced dominant societal values and discourses related to the age when drinking starts, the negative aspects of group pressure and responsibility for their own health. If these young people exceeded the implicit guidelines of their parents too much they

						felt guilty. Mainstream breakers didn't feel the same guilt as described above
**Lindelof et al., 2010**<br><br>**Food**	Field observation and semi-structured interviews	Yes – Bourdieu's theory of practice.	Denmark<br><br>Three-week summer camp which aimed to reduce adolescents' obesity by behavioural modification.	To explore obese adolescents' and their parents' views regarding the former's obesity.	28 obese adolescents aged 13-16.	Young people articulated large amounts of unhealthy food intake. They were ashamed of eating in such a way, hid it from parents and wished that current eating habits were healthier.
**Lunnay et al., 2011**<br><br>**Alcohol**	Focus groups	Yes – Bourdieu's theory of practice.	Australia<br><br>Metropolitan South Australia with familial backgrounds of varying levels of economic and cultural capital	Using Bourdieu's concept of symbolic capital situated within the framework of distinction to understand the social mechanisms that underpin young females' alcohol-related behaviours.	Young females aged 14–17 who have experience with drinking alcohol (number of participants unclear).	There is an inherent societal acceptance of drinking as a valued social activity (symbolic capital). There is value and power to heavy drinking as a habitual expectation.<br><br>Young females also drink for distinction and inclusion and can be

						alienated for drinking and exhibiting sexual behaviours.
**Madrigal et al., 2017**<br><br>**Food**	Focus groups	No theoretical perspective explicitly outlined.	Guatemala<br><br>Public and private schools in Guatemala City	To examine perceptions of "healthy weight" among adolescent girls; the perceived barriers to achieving or maintaining healthy weight; and the differences between private and public schools as a proxy for SES differences.	Twenty-eight girls aged 13-15 (12 from private and 16 from public schools) enrolled in grades 7 through 9.	"Healthy weight" was described as having proportionate bodies and feeling good about themselves.<br><br>Young girls agreed that a balanced diet and physical activity are the best ways to achieve and maintain a healthy weight.<br><br>A key barrier to healthy weight was the limited selection of healthy food options available at kiosks on school campus.
**McKay et al., 2011**<br><br>**Alcohol**	Focus group discussions	No theoretical perspective explicitly outlined.	Northern Ireland<br><br>25 post-primary schools (high schools) in the	To identify some of the attitudes and justifications for alcohol use in young people	25 focus groups.<br><br>227 young people from year 11 (age 15–16)	Young people were concerned with being caught drinking by their parents,

			greater Belfast area			and to a lesser degree getting home safely after drinking.<br><br>Alcohol use was so prevalent in their neighbourhoods and a part of everyday life that its misuse was not seen as particularly problematic.<br><br>Young people were concerned about looking foolish while drunk. They became more violent when drunk as well as more socially (and sexually) confidence and were more willing to take chances.
**Petrilli et al., 2014**<br><br>**Alcohol**	Focus groups	Yes – guided by Reception Analytic Group Interview Technique (RAGI).	Italy<br><br>Four secondary schools in the cities of Cosenza and Torino, representing both medium-	To explore how Italian adolescents perceive drinking in different situations. Video clips were used	22 focus groups; six interviewees were recruited per focus group; aged 15.	Young people drink because from, their point of view, everyone around them drinks.

			high and medium-low socio-economic environments.	to stimulate the discussion.		Students judged those who get drunk negatively, because they associate this behaviour with an individual shortcoming.
**Parder, 2018**<br><br>**Alcohol**	Small-group interviews	Yes – social practice theory.	Estonia<br><br>Three entire 8th grade classes from three schools in different parts of Estonia.	Investigating adolescents' non-drinking narratives in the context of social practise theory.	Adolescents aged 13–15.<br><br>15 interviews conducted with 49 students: 32 boys and 17 girls.	Alcohol has an important role in parties for adolescents and is used to build connections between friends. Those who drink at parties dismiss non-alcohol parties as childish.<br><br>The 'caretaker' was the non-drinker who took care of everyone else.
**Protudjer et al., 2010**<br><br>**Food**	Semi-structured, in-depth interviews	No theoretical perspective explicitly outlined.	Canada<br><br>Winnipeg, Manitoba	How children perceive healthful eating and physical activity.	boys (n=23) and girls (n=22) aged 11-12.	Children said that being healthy meant "you eat healthy, you only eat junk food once in a while, and you exercise lots.<br><br>Information about healthy

						eating was introduced in school by teachers and was reinforced at home by parents. However, it was still not a top-of-mind concern for them.<br><br>Young people described physical activity as being easier to do because "it's a lot more fun" than healthy eating.
**Rakhshanderou et al., 2014**<br><br>**Food**	Semi-structured interviews	No theoretical perspective explicitly outlined.	Tehran, Israel	To identify the determinants of fruit and vegetable consumption among Tehranian adolescents	31 adolescents aged 11-14: 52 % boys and 48% girls	Respondents eat fruits every day in their homes because their parents always provide fruits at home and they must obey their wishes.<br><br>Unhealthy junk food in school and home is a barrier preventing consumption of fruits and vegetables.

**Ridder et al., 2009**<br><br>**Food**	Focus groups	Yes – EnRG framework.	Netherlands<br><br>Seven lower vocational schools in the Zwolle region; a rural area in northeast Netherlands	To investigate the benefits, barriers and strategies of healthy eating and physical activity among adolescents	5 focus groups<br><br>37 second grade lower vocational students (20 girls, 17 boys) aged 12-14.	Adolescents find health and a healthy weight important and like having a choice when it comes to health behaviour. The choices they make, however, are often unhealthy, especially when related to food.<br><br>The risk perception of these adolescents is low. As long as they feel healthy, they feel no need to change their behaviour.<br><br>Parents are held responsible for providing opportunities for healthy behaviour.
**Romo-Avilés et al., 2018**<br><br>**Alcohol**	Focus groups and interviews	Yes – content analysis from a gender perspective.	Spain<br><br>13 secondary schools and youth	To analyse the way in which the gender system is interwoven with	10 focus group discussions and 30 in-depth interviews<br><br>96 males and females aged 14-17 who consumed alcohol at least once during the previous 12 months	Alcohol intake is strongly mediated by a social dimension -

			associations in Granada and Seville, two provinces of Andalusia in Southern Spain.	the meanings and motivations attributed by adolescents to alcohol consumption, mainly focussing on the experiences of the females.		adolescents want to lose inhibitions and feel good among and with their peers; the peer group formed a protective role.<br><br>Intoxication was a positive emotional state which enhances the development of social relationships. Drinking alone was regarded as problematic; it was associated with an adult pattern of alcohol consumption.
**Ronto et al., 2016**<br><br>**Food**	Focus groups	No theoretical perspective explicitly outlined.	Australia<br><br>16 schools in South East Queensland	Adolescents' perspectives of the importance of food literacy on their dietary behaviours.	15 focus groups (n=131); young people aged 12-17.	Adolescents reported being very interested in developing food skills such as food preparation but they had very limited opportunities due to lack of food literacy education in

						home and high school settings.<br><br>Adolescents believed that it was schools' and parents' responsibility to teach them food preparation and cooking skills.
**Samardzic et al., 2011**<br><br>**Alcohol**	Essay writing	No theoretical perspective explicitly outlined.	Croatia<br><br>First classes in medical high school	To investigate the drinking pattern of high school students, including the quantity of alcohol consumed as well as the reasons for it.	59 adolescents aged 14 or 15 years old	Adolescents reported that they drink alcohol to be happy, to relax, to be courageous in approaching the opposite sex, to fit into society, and to be popular.
**Sandberg and Skjælaaen, 2017**<br><br>**Alcohol**	Focus groups and interviews	No explicit theoretical approach outlined, however the authors describe a sociological approach to childhood	Norway<br><br>Seven 8th grade classes in different middle schools from three regions of Norway (both suburbs and urban settings).	To explore adolescents' views on alcohol and giving voice to their own associations, experiences, and opinions	29 focus group interviews and 32 interviews.<br><br>141 participants: 70 girls and 71 boys.	Adolescents are highly sceptical of drinking. They believe that people drink because they ''want to be cool'', because they are miserable, or because of group pressure.

						The perceived effects of alcohol consumption include getting drunk and addicted.<br><br>Those who drink alcohol are described as immature and marginalised.
**Scott et al., 2017**<br><br>**Alcohol**	Interviews	Yes – Bourdieu's theory of practice.	UK<br><br>North East England	To adopt Bourdieu's idea of social game-playing to enhance understanding of the drivers of mid-adolescent drinking practices	Adolescents aged 13–17 who were current users of alcohol at the time of the study (n=31).	Drinking practices were formed by friendships and family traditions, as well as culturally shaped through brand identification and alcohol marketing.<br><br>Adolescents needed to understand and adhere to distinct 'rules of play' regarding drinking. If not, they risked exclusion.
**Silva et al., 2015**	Semi-structured interviews	No explicit theoretical	Brazil	To analyse how adolescents	Forty adolescents aged 10-14.	Lunch was the most important

**Food**		approach outlined.	School in the State of Pernambuco	perceive healthy eating.		meal for adolescents.<br><br>Fruit consumption was mainly among adolescents who brought this snack from home or ate fruit at home.<br><br>Economic level and the lower cost of nutritionally inappropriate foods rich in sugar, fat and salt meant that young people favoured the consumption of these foods.
**Simonen et al., 2017**<br><br>**Alcohol**	Focus groups	Yes – applies the sociological concept of trust.	Finland<br><br>City-based	To understand how trust is manifested, maintained and challenged in teenagers' accounts of alcohol-related communication inside the family	8 focus groups; 54 adolescents aged 14–17.	Teenagers do not exclude their parents from alcohol-related discussion but expect rules, communication and authority from them.<br><br>Both being misleading and being open act as strategies that teenagers

						have created as a resource when trying to maintain a trustful relationship.
**Stead et al., 2011**<br><br>**Food**	Focus groups	No explicit theoretical approach outlined, however the authors describe a sociological approach consumption.	UK<br><br>North East England	To explore how young people use and interpret food and drink products, focusing in particular on one social context: the school packed lunch	12 focus groups (n=80); Adolescents aged 13-15.	Food choices carry meanings and judgements. Adolescents attach values and meanings to the food choices made by their peers. There are social risks to healthy eating.
**Stephens et al., 2015**<br><br>**Food**	Semi-structured interviews	Yes – grounded theory	Australia<br><br>Three co-educational Catholic secondary schools from socioeconomically disadvantaged suburbs in metropolitan and non-metropolitan regions of Victoria.	To identify potential strategies and preferred dissemination methods of nutrition promotion initiatives among socioeconomically disadvantaged adolescents	22 adolescents aged 12–15	Young people articulated the importance of: increasing awareness about healthy eating; greater cooking involvement; greater parental and peer support; frequent family meal participation; decreased availability of unhealthy foods in homes and schools.
**Stevenson et al., 2007**	Focus groups	No explicit theoretical	Ireland	To examine potential	12 focus groups; 73 adolescents aged 12–13	Whilst adolescents

**Food**		approach outlined.	Rural and urban second level schools; North and South	conceptual, physical, individual, developmental and social barriers to healthy eating.		have good knowledge of what is healthy, nutritional knowledge may not be the central motivation for food choice. There is a balance between healthiness and tastiness of foods as a determinant of food preference.
**Taylor, 2015**<br><br>**Food**	Interviews, focus groups and participant observation	No explicit theoretical approach outlined.	USA<br><br>Suburban, primarily middle class high school, located in the Southwest	To explore youth perceptions of school food, attitudes about overweight and obesity, and gendered food consumption behaviours within the broader context of competing popular discourses about the national "obesity epidemic."	50 participants (age not clear)	Adolescents criticized overweight people for a perceived lack of self-control. However, they reported feeling out of control with regard to their own food consumption behaviours at school.<br><br>Girls blamed themselves for eating "junk food" at school and engaged in "guilt talk" to

						mitigate feelings of guilt.<br><br>Young people felt overwhelmed with temptation to consume junk food all day at school because it was so pervasive.
**Townshend, 2013**<br><br>**Alcohol**	Interviews and focus groups	Yes – orientated by Bourdieu's concept of habitus and Gibson's work on affordances.	UK<br><br>Suburban neighbourhoods in England	To investigate the 'how' and 'where' of young people's engagement with both alcohol consumption and non-alcohol related leisure	50 young people aged 15-16.	Many participants admitted transgressing parental control and any family centred nature of alcohol consumption quickly became replaced by one that was focused on friendships and socialisation. Nevertheless, participants stated that friends did not unduly influence each other.
**Trujillo et al., 2015**<br><br>**Alcohol**	Semi-structured interviews	Yes – social learning theory.	Colombia<br><br>Middle school students (7th	To explore how adolescents learn about risk and behaviour	12 interviews.<br><br>160 adolescents aged 12-14.	Adolescents can identify several risks related to the

			and 8th graders) from two schools in Bogotá, in the localities of Kennedy and Santa Fe.	with regards to alcohol use in light of Social Learning Theory.		use of alcohol. However, this risk recognition does not appear to correlate with their drinking behaviour.
**Van Hoof et al., 2011**<br><br>**Alcohol**	Semi-structured interviews.	Yes – based on the critical incident technique (CIT).	Netherlands<br><br>Schools	How young adolescents make sense of alcohol experiences in their lives.	45 adolescents, aged 15 and 16.	Parents usually knew about the alcohol incident but they only knew the 'tip of the iceberg'.<br><br>Adolescents had the tendency of turning adverse consequences of alcohol use into positive overall evaluations.
**Watts et al., 2015**<br><br>**Food**	Photovoice methods followed by semi-structured interviews	Yes – phenomenological inquiry	Canada<br><br>Vancouver, British Columbia (BC)	To explore perceived factors that impede or facilitate healthful eating within the home environment among overweight/obese adolescents	22 overweight/obese adolescents aged 11–16.	Homes with availability of less healthful foods, where family members also liked to eat less healthful foods and where healthier foods were less abundant or inaccessible, were described as barriers to

						healthful eating.<br><br>Special occasions and time spent studying or in front of the screen were also viewed negatively.<br><br>Home cooked meals allowed young people to make healthier food choices; parental encouragement and restriction were helpful for some adolescents.
**Wills et al., 2008**<br><br>**Food**	Interviews	Yes – not one explicit theoretical perspective outlined, but rooted in sociological approaches to family, childhood and the role of food.	UK<br><br>Three schools in Scotland	To explore young teenagers' dietary worlds, looking at the social influences that emerged from participants' narratives about everyday food and eating practices	Adolescents aged 13-14 (n=36)	Food and eating practices were negotiated amidst complex family arrangements, reflecting both teenagers' and parents' lifestyles.<br><br>Teenagers could differentiate themselves from others

						through their food preferences and tastes and food consumption therefore helped shaped their identity.
**Wills et al., 2016**<br><br>**Food**	Focus groups, a semi-structured written exercise, lunchtime 'go-along' tours, and individual or group interviews	Yes - situated within a children's rights framework, underpinned by the sociology of childhood	UK<br><br>Secondary schools in Scotland	To highlight some of the factors that might influence young people to purchase food or drink within or beyond the school catering service	Adolescents aged 13-15. A range of methods used with pupils from 7 schools (no exact participant numbers provided).	Adolescents were content to purchase and eat food and drink within the school as it is convenient. However, they complained about the quality, taste, availability, portion size and price. Marketing and promotion of food and drink in schools was misleading in terms of taste.
**Witmer et al., 2011**<br><br>**Food**	Focus groups	Yes – grounded theory	USA<br><br>Southeast	To explore adolescent girls' attitudes toward leisure-time physical activity (LTPA), eating, physical health, and the perceived influences on health behaviours	28 adolescent girls in the 6th, 7th and 8th grade (11 Caucasian, 14 African-American, 3 Latina)	Four themes emerged regarding girls' preferences for and attitudes toward PA and nutrition: a) perceptions of health, b) family and food, c) social

				within a leisure context.		norms, and d) enjoyment.
**Yoon et al., 2015**<br><br>**Alcohol**	Focus groups	Yes – grounded theory.	Hong Kong<br><br>Schools	To investigate the social and cultural contexts that shape the drinking trajectories of Chinese adolescents (how young people learn to drink, how they perceive their own alcohol use and what factors influence how they make decisions about the consumption (or not) of alcohol)	22 focus groups (n=111); adolescents aged 14-17	Motivations for drinking are to promote conviviality, sociability and camaraderie. Drinkers and non-drinkers had the same friendship networks.<br><br>Adolescents had a high degree of self-regulation when drinking.<br><br>Underage drinking was a normal and accepted part of social lives provided that it is kept under control.
